# Effect of Denervation on XBP1 in Skeletal Muscle and the Neuromuscular Junction

**DOI:** 10.3390/ijms23010169

**Published:** 2021-12-24

**Authors:** Lisa A. Walter, Lauren P. Blake, Yann S. Gallot, Charles J. Arends, Randall S. Sozio, Stephen M. Onifer, Kyle R. Bohnert

**Affiliations:** 1 Department of Kinesiology, St. Ambrose University, Davenport, IA 52803, USA; walterlisaa@sau.edu (L.A.W.); blakelaurenp@sau.edu (L.P.B.); 2 LBEPS, Univ Evry, IRBA, Université Paris Saclay, 91025 Evry, France; yann.gallot@univ-evry.fr; 3 Palmer Center for Chiropractic Research, Palmer College of Chiropractic, Davenport, IA 52803, USA; charles.arends@uconn.edu (C.J.A.); sozior00@yahoo.com (R.S.S.); stephenmonifer@gmail.com (S.M.O.)

**Keywords:** skeletal muscle atrophy, denervation, ER Stress, UPR, neuromuscular junction

## Abstract

Denervation of skeletal muscle is a debilitating consequence of injury of the peripheral nervous system, causing skeletal muscle to experience robust atrophy. However, the molecular mechanisms controlling the wasting of skeletal muscle due to denervation are not well understood. Here, we demonstrate that transection of the sciatic nerve in Sprague–Dawley rats induced robust skeletal muscle atrophy, with little effect on the neuromuscular junction (NMJ). Moreover, the following study indicates that all three arms of the unfolded protein response (UPR) are activated in denervated skeletal muscle. Specifically, ATF4 and ATF6 are elevated in the cytoplasm of skeletal muscle, while XBP1 is elevated in the nuclei of skeletal muscle. Moreover, XBP1 is expressed in the nuclei surrounding the NMJ. Altogether, these results endorse a potential role of the UPR and, specifically, XBP1 in the maintenance of both skeletal muscle and the NMJ following sciatic nerve transection. Further investigations into a potential therapeutic role concerning these mechanisms are needed.

## 1. Introduction

Skeletal muscle is considered an essential tissue involved in metabolism, thermoregulation, protection of some internal organs, postural maintenance, respiration, and locomotion. This organ is richly supplied by blood arterioles allowing its nourishment, and is innervated through the peripheral nervous system (PNS) by a peculiar site, namely the neuromuscular junction (NMJ). The NMJ is a highly specialized synaptic connection between the terminal end of a motor nerve and its skeletal muscle fiber. It transduces the excitatory electrical impulse generated by the motor neuron into electrical activity in the skeletal myofiber, causing ultimately its contraction. Functional innervation has a crucial role during the development and maturation of skeletal muscle and is absolutely required for its postnatal growth and maintenance [1,2]. In the complete absence of motor innervation or following injury of the PNS, skeletal muscle experiences an inescapable atrophy, leading to a marked decrease in contractile force production [3]. Progressive acquired or hereditary neuromuscular diseases, such as amyotrophic lateral sclerosis (ALS), the axonal form of Guillain-Barré syndrome, spinal muscular atrophy (SMA), or Charcot-Marie-Tooth disease (CMT) are characterized by a motoneuron loss. This neuromuscular failure is also associated with sarcopenia, neoplasia, cancer surgery, viral infections, and trauma. The consequences of skeletal muscle denervation are well-documented and engender a loss of motor function, weakness and fatigue, respiratory failure, and even mortality [4,5,6,7]. However, despite the prevalence and severity of denervation-induced skeletal muscle atrophy, the molecular mechanisms that regulate the loss of skeletal muscle mass is unclear.

Most eukaryotic cells possess an extensive network of membranous tubules and flattened sacs within the cytoplasm, named the endoplasmic reticulum (ER). This dynamic cellular organelle serves several roles, including calcium storage, carbohydrate, and lipid metabolism, and manages the correct folding, structural maturation, and transport of newly synthesized secretory and transmembrane proteins [8,9,10,11,12]. In addition, skeletal myofibers comprise a specialized form of ER referred to as sarcoplasmic reticulum (SR). The SR is a differentiated domain of the skeletal muscle ER [13], derived from it by proliferation and differentiation and mainly dedicated to calcium homeostasis [14]. In particular, the SR controls the regulated release of calcium ions (Ca^2+^) into the cytoplasm to trigger a skeletal muscle contraction [15]. While protein processing in the ER normally occurs in an orderly fashion, several physiological and pathological stimuli can alter normal ER homeostasis, eventually inducing an accumulation of unfolded and misfolded proteins in the ER lumen [8,9,10,11,12]. To prevent these disturbances in proteostasis, heuristically known as ER stress, eukaryotic cells elicit an adaptive intracellular mechanism, the unfolded protein response (UPR). The UPR causes the expression and the increased production of ER chaperones in order to clear damaged proteins and to restore normal ER homeostasis [16]. The initiation of the UPR depends on three key signaling cascades induced by the ER transmembrane transducers: activating transcription factor-6 (ATF6), inositol-requiring protein (IRE) 1α, and protein kinase R (PKR)-like endoplasmic reticulum kinase (PERK) [8,17,18].

ATF6, IRE1α, and PERK are protein sensors that are maintained in an inactive state through binding to the ER resident molecular chaperone 78 kDa glucose-regulated protein (GRP78), which is also referred to as binding immunoglobulin protein (BiP) or heat shock protein family A member 5 (HSPA5). However, under stressful conditions, the interaction of GRP78/BiP/HSPA5 with the ER sensors is relieved, freeing them to initiate the UPR. PERK, also known as eukaryotic translation initiation factor 2-alpha kinase 3 (EIF2AK3), is activated by trans-autophosphorylation and oligomerization after the release of GRP78/BiP/HSPA5. Then, PERK′s cytosolic active kinase domain directly phosphorylates the α-subunit of eukaryotic translation initiation factor 2-alpha (eIF2α) and induces an increased translation of activating transcription factor-4 (ATF4), which stimulates a key regulator of ER stress-induced apoptosis, the proapoptotic transcription factor named CCAAT-enhancer-binding protein homologous protein (CHOP) [8,19,20]. Additionally, the PERK-eIF2α signaling pathway mediates the resolution of the UPR and the restoration of the protein synthesis upon stress recovery through activation of protein phosphatase 1 regulatory subunit 15A (PPP1R15A), also known as growth arrest, and DNA damage-inducible protein 34 (GADD34), which dephosphorylates p-eIF2α [21]. IRE1α undergoes conformational change, autophosphorylation, and oligomerization and becomes activated upon ER stress, triggering its endoribonuclease (RNase) activity. This induces the splicing of a 26-base intron from X-box-binding protein 1 (XBP1) mRNA [22]. Spliced XBP1 (sXBP1) is a potent transcription factor and transcriptionally reprograms UPR target genes, including ER chaperones, to restore ER homeostasis [23]. Lastly, upon the accumulation of misfolded proteins in the ER, GRP78/BiP/HSPA5 dissociation allows ATF6 activation and triggers relocation of ATF6 from the ER to the Golgi apparatus, where it is cleaved by proteases [24]. Following this proteolytic cleavage, the resulting N-terminal fragment of ATF6 translocates to the nucleus and activates a transcriptional program to resolve ER stress [22].

The progress made in recent years has greatly highlighted the role of UPR components in maintaining muscle mass in many catabolic conditions, especially denervation [25,26,27,28,29,30,31]. In surgically denervated wild-type mice, as well as in a knock-in mouse model of spinal and bulbar muscular atrophy (SBMA), AR113Q mice, cellular indicators of ER stress and the UPR, including CHOP, are found to be upregulated in skeletal muscle [25]. Additionally, the genetic deletion of CHOP enhanced the muscle atrophy experienced after denervation and in the AR113Q mice. ER stress and the coping signaling pathways of the UPR have also been induced in the superoxide dismutase 1 (SOD1) models of ALS [30,32]. Precisely, the IRE1α and PERK branches of the UPR were triggered in early, pre-symptomatic stages and continued to increase as the disease progressed [30]. Interestingly, induction of the macroautophagy (referred to here as autophagy) following XBP1 knockdown was described to ameliorate the pathology of mutant SOD1 transgenic mice. Researchers showed that the absence of XBP1 stimulated the autophagic–lysosomal pathway, which promoted the degradation of mutant SOD1, resulting in clearance of the aggregates [32]. More recently, deletion of XBP1 in skeletal muscle was found to have beneficial effects on skeletal muscle wasting [29]. These studies provide an early understanding of the role of ER stress and UPR in skeletal muscle regulation following denervation. However, how ER stress disrupts the skeletal muscle and the NMJ homeostasis is not well-known.

Using sciatic nerve transection inducted muscle atrophy in Sprague–Dawley (SD) rats, we investigated what role ER stress and the UPR has in the maintenance of the NMJ. Our morphometry results demonstrated that the sciatic nerve transection leads to a robust skeletal muscle atrophy in SD rats by day 14. Moreover, sciatic nerve transection leads to the accumulation of ATF4 and ATF6 in the cytoplasm of skeletal muscle. Interestingly, denervation promotes the accumulation of XBP1 specifically in the nuclei of the muscle and surrounding cells. Finally, our results feature that XBP1 and not ATF4 or ATF6 is localized at the NMJs that lack muscular input.

## 2. Results

### 2.1. Transection of the Sciatic Nerve for 14 Days Induces a Robust Skeletal Muscle Atrophy in Sprague–Dawley Rats

**Figure 1:**
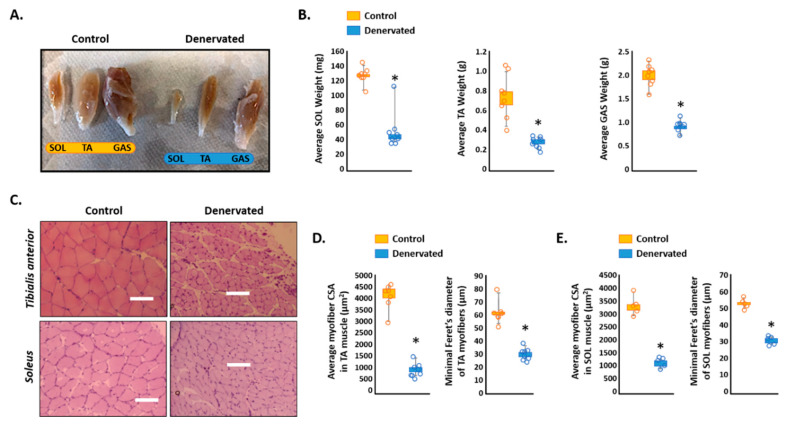
Effect of sciatic nerve transection on skeletal muscle maintenance in Sprague–Dawley (SD) rats. Eight male rats had their sciatic nerves ligated on one side of the body. After 14 days, the SD rats were sacrificed and perfused. (**A**) Visual depiction of the control and denervated leg muscles, Scale bar = 100 μm. (**B**) Average wet weight of *soleus* (SOL), *tibialis anterior* (TA) and *gastrocnemius* (GAS). (**C**) Hematoxylin and Eosin (H&E) stain of the TA and SOL of control and denervated muscle. Scale = 100 μm. (**D**,**E**) The average cross-sectional area (CSA) and minimal Feret′s diameter of the control and denervated TA and SOL muscles. * *p* < 0.05, values significantly different from the control mice by a paired two-tailed *t*-test.

Sciatic nerve transection is a common procedure to induce skeletal muscle atrophy [3,33,34]. We first set out to confirm that our model of sciatic nerve transection was robust enough to induce skeletal muscle atrophy after 14 days. Figure 1A provides a visual representation of lower extremity muscles on both the control and the denervated side. The wet weight of the *soleus* (SOL), *tibialis anterior* (TA), and *gastrocnemius* (GAS) muscles all showed significant reductions after sciatic nerve transection (Figure 1B). Moreover, the average CSA and minimal Feret′s diameter of both the TA and SOL muscles were significantly decreased on the denervated side as compared to the control side (Figure 1C–E).

**Figure 2:**
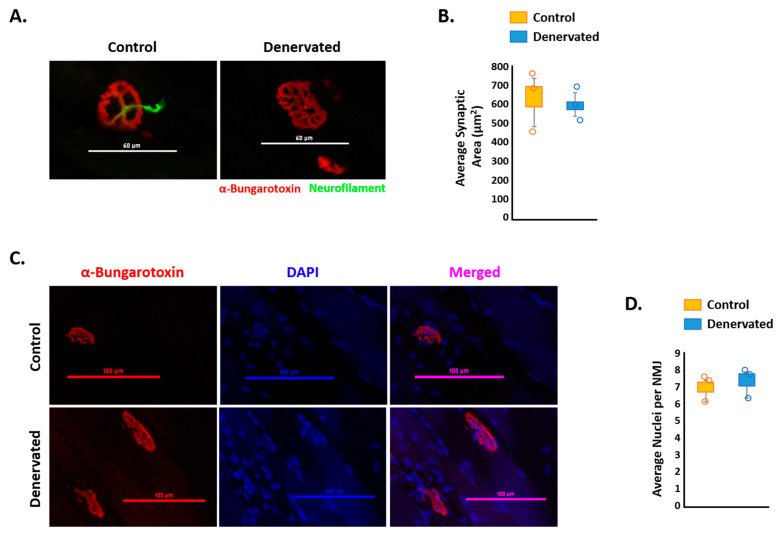
Effect of sciatic nerve transection on neuromuscular junction (NMJ) maintenance. (**A**) Representative images of the NMJ in the control and denervated GAS muscles immunostained with a snake toxin with a high affinity for AChRs (α-bungarotoxin-tetramethylrhodamine) and neurofilament. Scale bar = 60 μm. (**B**) Average synaptic area in the control and denervated GAS muscles. (**C**) Representative images of the NMJ using snake toxin with a high affinity for AChRs and nuclear staining (DAPI). Scale bar = 100 μm. (**D**) Average nuclei per NMJ in the control and denervated GAS muscles. Values significantly different from the control mice by a paired two-tailed *t*-test.

Loss of motor neuron innervation in the GAS muscle was further shown through immunostaining of the acetylcholine receptor AChR (α-Bungarotoxin-tetramethylrhodamine) and neurofilament (Figure 2A). Intriguingly, the loss of motor neuron input did not have any significant effect on the synaptic area (Figure 2B). Additionally, the sciatic nerve transection did not have any significant effect on the number of nuclei per NMJ (Figure 2C,D). This data revealed that our model of sciatic nerve transection was sufficient to produce noticeable skeletal muscle atrophy after 14 days of denervation. However, the denervation had no significant effect on the structure of the NMJ.

### 2.2. Effect of Sciatic Nerve Transection on Expression of the Unfolded Protein Response in Skeletal Muscle

**Figure 3:**
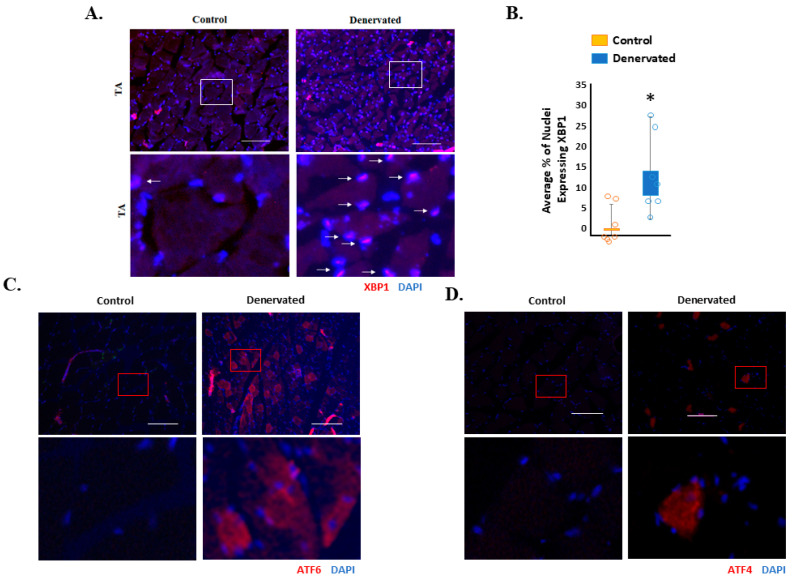
Effect of transection of the sciatic nerve on expression of the unfolded protein response (UPR) in skeletal muscle. (**A**) Representative images of the control and denervated TA muscles immunostained against XBP1 and DAPI. Scale bar = 100 μm. (**B**) Average percentage of nuclei expressing XBP1 in the control and denervated TA muscles. (**C**) Representative images of the control and denervated TA muscles immunostained against ATF6 and DAPI. (**D**) Representative images of the control and denervated TA muscles immunostained against ATF4 and DAPI. * *p* < 0.05, values significantly different from the control mice by a paired two-tailed *t*-test.

Recent studies have identified that ER stress and the UPR are activated in denervated skeletal muscle [29]. However, the location of the upregulation of these molecules is not yet known. Figure 3A provides a visual representation of the TA muscle immunostained with antibodies against XBP1 and DAPI. It is worth noting that denervation of the TA muscle led to an increase in the colocalization of XBP1 and DAPI (Figure 3B). In contrast, transection of the sciatic nerve caused an accumulation of both ATF4 and ATF6 in the cytoplasm of the skeletal muscle (Figure 3C,D). However, there was no colocalization of either molecule with DAPI in the control or denervated TA. This data indicates that, although all three major transcription factors of the UPR are activated following sciatic nerve transection, only XBP1 produced a colocalization with the nuclei.

### 2.3. Effect of Sciatic Nerve Transection on Expression of the Unfolded Protein Response in the Neuromuscular Junction

**Figure 4:**
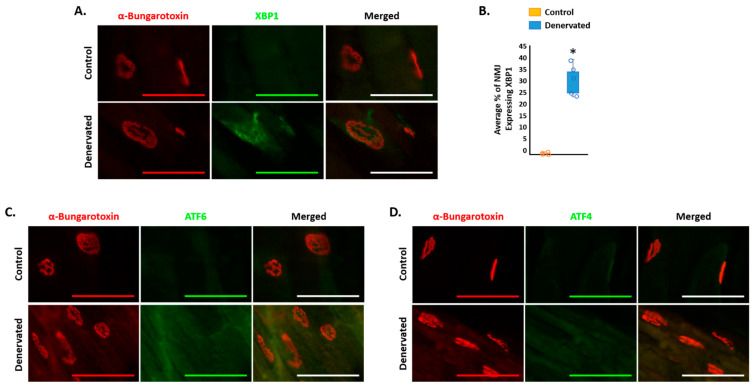
Effect of transection of the sciatic nerve on expression of the unfolded protein response (UPR) in the neuromuscular junction (NMJ). (**A**) Representative images of the control and denervated GAS muscles immunostained against XBP1 and with a snake toxin that has a high affinity for AChRs (α-bungarotoxin-tetramethylrhodamine). Scale bar = 100 μm. (**B**) Average percentage of NMJ expressing XBP1 in control and denervated muscle. *N* = 6/group. (**C**) Representative images of the control and denervated GAS muscles immunostained against ATF6 and with a snake toxin that has a high affinity for AChRs (α-bungarotoxin-tetramethylrhodamine). Scale bar = 100 μm. (**D**) Representative images of the control and denervated GAS muscles immunostained against ATF4 and with a snake toxin that has a high affinity for AChRs (α-bungarotoxin-tetramethylrhodamine). Scale bar = 100 μm. * *p* < 0.05, values significantly different from the control mice by a paired two-tailed *t*-test.

Upon nerve transection, NMJ undergo destabilization and fragmentation [33,34]. However, the mechanisms that lead to this event are still yet to be fully understood. Figure 4A–D depicts the NMJ of the control and denervated GAS muscles through immunostaining of the AChR. Interestingly, transection of the sciatic nerve induced colocalization of XBP1 with the AChR (Figure 4A,B). In contrast, transection of the sciatic nerve did not induce such colocalization of the AChR with ATF4 or ATF6 (Figure 4C,D). These results suggest a potential role of XBP1 in destabilization of the NMJ following transection of the sciatic nerve.

## 3. Discussion

The ER stress and UPR have recently seen increased interest in their role in the maintenance of skeletal homeostasis. It is worth mentioning that UPR has been shown to have differential effects on skeletal muscle depending on the context [15,31]. Recently, researchers have determined that ER stress and UPR are triggered in skeletal muscle following sciatic nerve injury [29]. However, the role of the UPR in skeletal muscle maintenance following neurological input has yet to be elucidated. In the present study, we provided evidence that all three arms of the UPR were activated in skeletal muscle following sciatic nerve injury in SD male rats. Interestingly, XBP1, the major transcription factor downstream of IRE1α, was also proven to be induced around the NMJ following denervation.

Recent studies have further investigated the role of XBP1 in skeletal muscle following denervation. Intriguingly, XBP1 deletion in SOD1-mutated mice induced autophagy, leading to the degradation of the mutant SOD1 aggregates [32]. More recently, skeletal muscle specific deletion of XBP1 had beneficial effects on skeletal muscle wasting following a sciatic nerve injury [29]. The current study demonstrated that XBP1 was specifically induced in the nuclei of skeletal muscle following a sciatic nerve injury (Figure 2A,B). As a transcription factor, XBP1 migrates from the cytoplasm of the cell to the nuclei when spliced by IRE1α to induce the transcription of various genes [18]. The presence of XBP1 in the nuclei of skeletal muscle suggests that the molecule is in the active form and effecting the transcription of its target genes. However, the possibility also exists that XBP1 is also expressed within the nuclei of non-muscular cells residing between fibers. Future studies would need to be completed to elucidate this possibility.

ATF4 and ATF6 mRNA levels were recently highlighted to be upregulated in skeletal muscle following sciatic nerve injury [29]. Moreover, the deletion of ATF4 had no effect on rescuing skeletal muscle following muscle denervation [33]. Interestingly, our results demonstrated that both ATF4 and ATF6 were elevated in the cytoplasm of skeletal muscle following a sciatic nerve injury. Both ATF4 and ATF6 have the ability to translocate to the nucleus to act as a transcription factor. Thus, the accumulation in the cytoplasm and not the nuclei of skeletal muscle indicates that, while these molecules may be induced in skeletal muscle following nerve injury, neither ATF4 and ATF6 act as active transcription factors.

NMJ destabilization and fragmentation occurs after denervation and in NMJ diseases [34,35,36]. The current study revealed that, following sufficient denervation (Figure 2A), there is no change in NMJ size (Figure 2B–D). However, Figure 1 reported that the injury to the sciatic nerve was indeed sufficient to induce robust atrophy. Thus, these results suggested that 14 days could have been too premature to visualize any change in NMJ size or stabilization. Although the current results described no effect yet on the NMJ, it is interesting to mention that an activation of XBP1 was observed specifically around the NMJ (Figure 4A,B). There was no such colocalization with other transcription factors of the UPR, ATF4 or ATF6 (Figure 4C,D). Thus, these results could demonstrate that XBP1 activation in the NMJ may precede the destabilization of the NMJ. Intriguingly, at this moment, it is unknown whether this colocalization is related to the expression in the skeletal muscle or potentially to the synaptic nuclei. Moreover, these results do not furnish evidence that XBP1 is beneficial or detrimental to the NMJ. Potentially, XBP1 may be active to prevent the destabilization of the NMJ. However, prolonged activation of XBP1 may provide adverse effects to the NMJ, similar to what is seen in skeletal muscle [27]. Additional studies of longer duration, as well as using pharmaceuticals, would need to be utilized to determine the effect that XBP1 has on NMJ stability following injury. It is likely that stimulation of the UPR and, specifically, XBP1 could be beneficial during denervation-induced atrophy. Moreover, the current study did not investigate the potential therapeutic effect of the inhibition of the UPR or XBP1 in NMJ diseases, such as ALS, where denervation has not yet occurred. Indeed, future studies genetically deleting XBP1 specifically in skeletal muscle during NMJ diseases, such as in ALS, could be essential in further understanding the mechanisms of those disorders and potential therapeutic effects.

In summary, our study provides initial evidence that ER stress and UPR may be involved in atrophy caused by injury to the sciatic nerve. In addition, this study indicates that XBP1 could play a role in the control of the destabilization of the NMJ following injury to the nerve. However, whether XBP1 is promoting destabilization of the junction or is helping to avert destabilization is not yet known. Although preliminary, these studies show the potential therapeutic effect of inhibition of the UPR on the maintenance of skeletal muscle in NMJ diseases.

## 4. Materials and Methods

### 4.1. Animals

Eight male Sprague–Dawley (SD) rats (Envigo, Indianapolis, IN, USA) were singly housed on Tek-Fresh bedding (Envigo) with environmental enrichment (PVC T-tubes and chew toys) and maintained in a 12:12-h light–dark (LD) cycle (lights on 7:00 a.m.–7:00 p.m.). Access to 16% rodent diet (Envigo) and tap water was provided ad libitum. SD rats were acclimatized to their housing environment at least 1 week prior to experimentation. All methods were approved by the Palmer College of Chiropractic Institutional Animal Care and Use Committee (IACUC).

### 4.2. Spared Nerve Injury

As described previously [37], the SD rats were anesthetized in an induction chamber with isoflurane (2.5%, Butler Schein Animal Health, Dublin, OH, USA) in oxygen (1 L/min) for 3 min. Ophthalmic ointment (Altaire Pharmaceutical Inc., Aquebogue, NY, USA) was placed over the eyes. The antibiotic Baytril (10 milligrams/kilogram, Bayer Healthcare LLC, Shawnee Mission, KS, USA) was injected subcutaneously. The lateral left hindlimbs were shaved between the hip and the knee, then wiped with povidone-iodine (Well @ Walgreens, Deerfield, IL, USA) and isopropyl alcohol (Well @ Walgreens) 3 times. Each rat was placed prone over a draped homeothermic blanket (Harvard Apparatus Inc., Holliston, MA, USA). Using the aseptic technique, an incision was made in the skin over the left femur. The fascia between the biceps femoris and gluteus superficialis muscles was cut. The muscles were retracted to expose the sciatic nerve. The tibial and common peroneal nerve branches were ligated with 6–0 silk (Covidien, Mansfield, MA, USA) and then transected 1 mm distal and proximal to the ligation. Care was taken to avoid damage to the sural nerve branch. The muscles and fascia were closed with 5–0-coated VICRYL* sutures (Ethicon, Somerville, NJ, USA). The skin was closed with 11-millimeter Michel wound clips (Fine Science Tools, Foster City, CA, USA). Bacitracin zinc ointment (Well @ Walgreens) was applied to the skin wound. Postoperatively, the SD rats were placed in clean cages with fresh bedding and on heating pads for recovery and then into the housing room. Twice-daily physical examinations and once-daily weighing were performed during the first week post-surgery. Seven days postoperatively, the wound clips were removed under isoflurane anesthesia. Afterwards, once-daily physical examinations and weighing were done.

### 4.3. Histology and Morphometric Analysis

The SD rats were euthanized after 14 days with Fatal-Plus® (0.88 milliliters/kilogram, intraperitoneal, Vortech Pharmaceuticals Ltd., Dearborn, MI, USA), followed by thoracotomy [38]. Transcardiac perfusion was quickly performed with a Masterflex® L/S variable speed drive pump (flow rate = 130 milliliters/minute, Cole-Parmer, Vernon Hills, IL, USA). Room temperature 0.1-M phosphate-buffered saline (200 milliliters, PBS, Fisher Scientific, Hanover Park, IL, USA) containing 0.25% heparin (1000 Units/milliliter, Sagent Pharmaceuticals, Schaumburg, IL, USA) was followed by cold 10% buffered formalin phosphate (300 milliliters, Fisher Scientific) [39].

The *tibialis anterior* (TA), *soleus* (SOL), and *gastrocnemius* (GAS) muscles were harvested and weighed. Next, 10-μm-thick transverse cryosections were prepared and stained with hematoxylin and eosin (H&E) dye (Thermo Fisher Scientific, Rockford, IL, USA), as previously described [26,27]. The sections were examined and imaged using an Eclipse TE 2000-U microscope equipped with a Digital Sight DS-Fi1 camera (Nikon, Tokyo, Japan). The myofiber cross-sectional area (CSA) and minimal Feret′s diameter in the H&E-stained TA or SOL muscle sections were estimated using ImageJ software (NIH, Bethesda, MD, USA) by analyzing ~500 myofibers in each section in a blinded fashion.

### 4.4. Immunostaining

First, 10-μm-thick transverse cryosections of TA muscles were blocked for an hour with 2% goat serum (Thermo Fisher Scientific Life Sciences, Waltham, MA, USA) in PBS (Fisher Scientific), followed by incubation for 1 h with monoclonal antibodies against type XBP1 (PA5-27650, Invitrogen, Thermo Fisher Scientific Life Sciences, Waltham, MA, USA), ATF6 (PA1-16730, Invitrogen), and ATF4 (PA5-68802, Invitrogen). The secondary antibody used was goat anti-rabbit IgG (H + L) Alexa Fluor 555 (A32727, Invitrogen). The sections were washed thrice with PBS and stained with corresponding secondary antibodies overnight. After the secondary antibodies were removed, the slides were washed thrice and incubated with 4′,6-diamidino-2-phenylindole dihydrochloride (DAPI, MBD0015, Sigma-Aldrich, St. Louis, MO, USA) for 3 min. Following DAPI incubation, the slides were mounted using a fluorescent medium (Southern Biotech, Birmingham, AL, USA). The sections were visualized, the fluorescence was captured with an Eclipse Ci microscope using Nikon NIS-Elements Advanced Research Imaging Software (V 4.13, Nikon, Tokyo, Japan) run on a HP Z220 workstation (Hewlett-Packard, Palo Alto, CA, USA), and the images were analyzed using ImageJ software (NIH). The percentage of XBP1 colocalizing with DAPI-stained nuclei was analyzed for 500–750 nuclei per muscle.

Similar protocols were used to visualize the NMJ. In contrast, 40-μm-thick longitudinal cryosections of the gastrocnemius muscle were blocked with 2% goat serum in PBS (Fisher Scientific), followed by incubation for 1 h with monoclonal antibodies against type XBP1 (PA5-27650, Invitrogen, Thermo Fisher Scientific Life Sciences, Waltham, MA, USA), ATF6 (PA1-16730, Invitrogen), and ATF4 (PA5-68802, Invitrogen). The secondary antibody used was goat anti-rabbit IgG Alexa Fluor 488. The sections were washed thrice with PBS and stained with corresponding secondary antibodies overnight. After the secondary antibodies were removed, the slides were washed thrice and incubated with a snake toxin that has a high affinity for AChRs (α-bungarotoxin-tetramethylrhodamine, Sigma-Aldrich, T0195) for 1 h. Following AChR incubation, the slides were washed and incubated with DAPI for 3 min. Following DAPI incubation, the slides were mounted using a fluorescent medium (Southern Biotech). The sections were visualized, the fluorescence was captured with an Eclipse Ci microscope using Nikon NIS-Elements Advanced Research Imaging Software (V 4.13, Nikon) run on a HP Z220 workstation (Hewlett-Packard), and the images were analyzed using ImageJ software (NIH). Fifteen to thirty NMJ were analyzed for each muscle for the percentage of colocalization between the NMJ and the protein of interest.

### 4.5. Statistical Analyses

The results were expressed as box and whisker plots, with the box comprised of the first, second, and third quartiles and the lower and upper whiskers corresponding to the minimum and maximum values, respectively, to display the entire range of data. Statistical analyses between two groups used paired two-tailed Student′s *t*-tests to compare the quantitative data populations with normal distribution and equal variance. A value of *p* < 0.05 was considered statistically significant unless specified otherwise for comparisons made between two groups.

## Data Availability

Data from this study is secured in a password protected computer.
