# Correction: Walter et al. Effect of Denervation on XBP1 in Skeletal Muscle and the Neuromuscular Junction. *Int. J. Mol. Sci.* 2022, *23*, 169

**DOI:** 10.3390/ijms27114768

**Published:** 2026-05-26

**Authors:** Lisa A. Walter, Lauren P. Blake, Yann S. Gallot, Charles J. Arends, Randall S. Sozio, Stephen M. Onifer, Kyle R. Bohnert

**Affiliations:** 1 Department of Kinesiology, St. Ambrose University, Davenport, IA 52803, USA; walterlisaa@sau.edu (L.A.W.); blakelaurenp@sau.edu (L.P.B.); 2 LBEPS, Univ Evry, IRBA, Université Paris Saclay, 91025 Evry, France; yann.gallot@univ-evry.fr; 3 Palmer Center for Chiropractic Research, Palmer College of Chiropractic, Davenport, IA 52803, USA; charles.arends@uconn.edu (C.J.A.); sozior00@yahoo.com (R.S.S.); stephenmonifer@gmail.com (S.M.O.)

In the original publication [1], there was a mistake in Figure 3C,D as published. These images are immunohistochemical stains of the molecules ATF4 and ATF6 along with the nuclear stain DAPI. These images displayed how both ATF4 and ATF6 localized in the cytoplasm of the muscle after denervation of the sciatic nerve. This is different than XBP1, which localized in the nuclei. Unfortunately, in organizing the images during preparation an error was made where the images displayed for the ATF6 stain were the same displayed for the ATF4 stain. This occurred during this preparation phase due to a decision to change the order in which the stains were presented. After being made aware of the error, the authors confirmed the images displayed in the manuscript were the ATF6 stain not the ATF4 stain. The authors then added the correct images for ATF4 in Figure 3D. The corrected Figure 3C,D appear below. The authors state that the scientific conclusions are unaffected. This correction was approved by the Academic Editor. The original publication has also been updated.

## Figures and Tables

**Figure 3:**
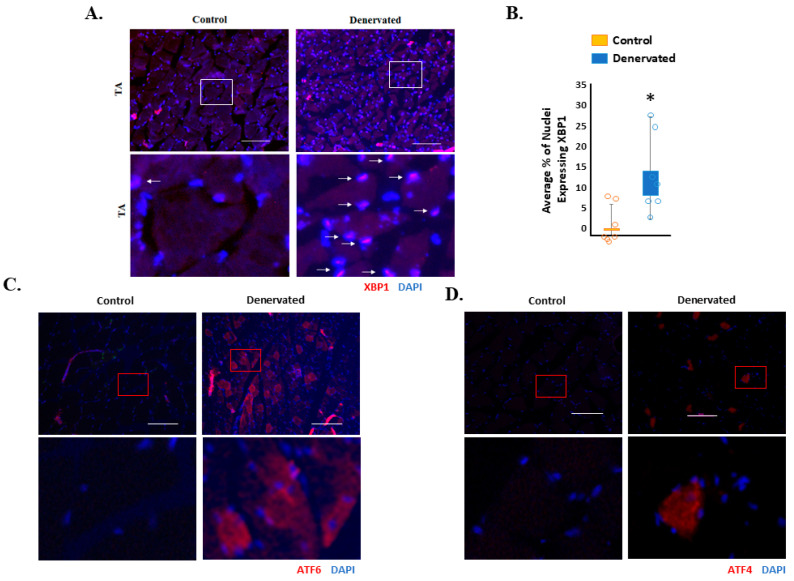
Effect of transection of the sciatic nerve on expression of the unfolded protein response (UPR) in skeletal muscle. (**A**) Representative images of the control and denervated TA muscles immunostained against XBP1 and DAPI. Scale bar = 100 μm. (**B**) Average percentage of nuclei expressing XBP1 in the control and denervated TA muscles. (**C**) Representative images of the control and denervated TA muscles immunostained against ATF6 and DAPI. (**D**) Representative images of the control and denervated TA muscles immunostained against ATF4 and DAPI. * *p* < 0.05, values significantly different from the control mice by a paired two-tailed *t*-test.
